# EXPLORING HOW AGE PREDICTS OUTNESS AND INTERNALIZED HOMOPHOBIA IN A LIFESPAN SAMPLE OF SEXUAL MINORITIES

**DOI:** 10.1093/geroni/igz038.1115

**Published:** 2019-11-08

**Authors:** Michael T Vale, Paige M Pasta, Toni L Bisconti

**Affiliations:** University of Akron, Akron, Ohio, United States

## Abstract

Minority Stress Theory posits that specific minority stressors, such as internalized homophobia and outness, predict negative health outcomes in sexual minorities. There has been substantial work in addressing these stressors in young adult samples; however, less in known about older adults. Older sexual minorities were socialized in a time in which same-sex relations were considered deviant and illegal, and therefore, have been exposed to a lifetime of marginalization. Although there is evidence that minority stressors negatively impact health in older adults, many studies exclude a complete lifespan sample. The goal of this study was to collect a sample of individuals in same-sex relationships ranging in age from 18-80 (N = 228, M = 40.93, SD = 15.87) and examine whether age correlates with outness and internalized homophobia. We found that older participants had higher degrees of outness (r = .21, p < .01) and less internalized homophobia (r = -.20, p < .01) resulting in less overall minority stress. We also analyzed different social resources that might explain the age-related decrease in minority stress and found that age was related to higher self-esteem (r = .24, p < .01). We tested whether self-esteem moderated the direct relationships between age and the minority stressors and found a significant interaction for internalized homophobia (B = .0175, p < .01), but not outness. Our findings provide support that older sexual minorities report less minority stress and more research is needed to explain what promotes these trends.

